# Supplementary figures and images for: Standardized Method for the Assessment of Behavioral Responses of Zebrafish Larvae
Source: Biomedicines. 2021 Jul 24;9(8):884. doi: 10.3390/biomedicines9080884 (PMC8389650; doi:10.3390/biomedicines9080884)

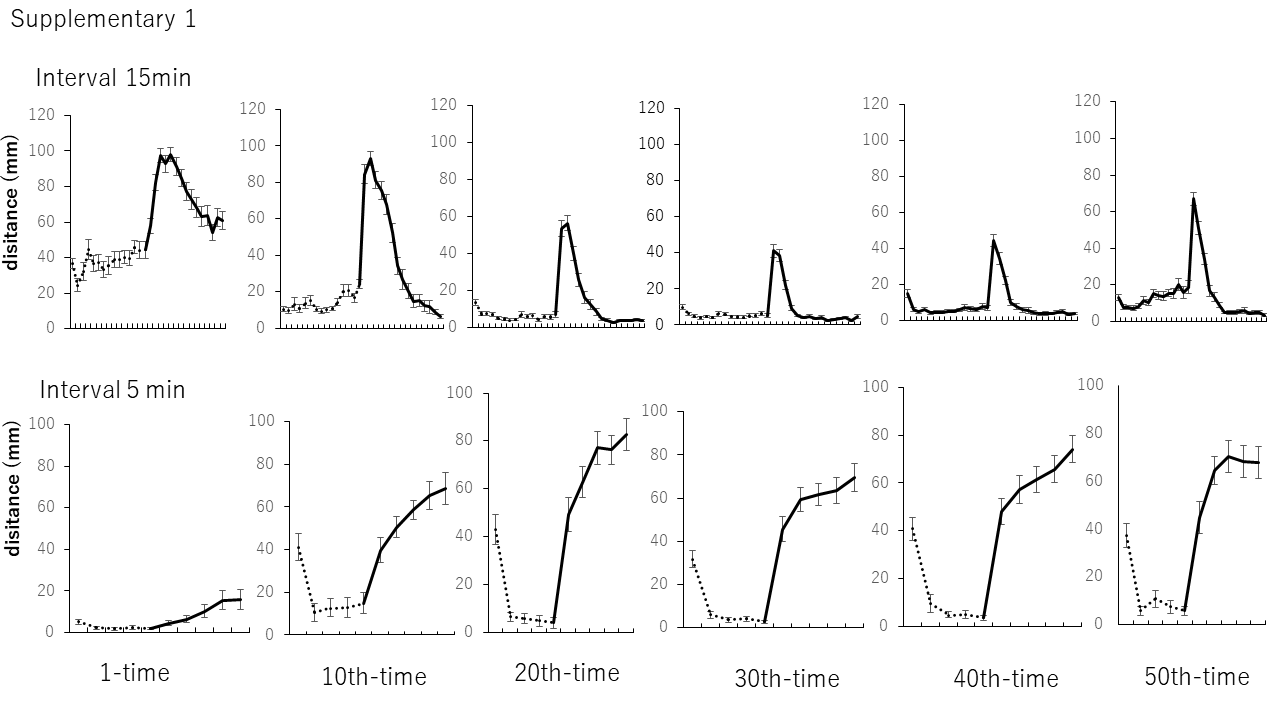

Supplement: Supplementary file 1 [file biomedicines-09-00884-s001.zip › Figure S1.TIF]

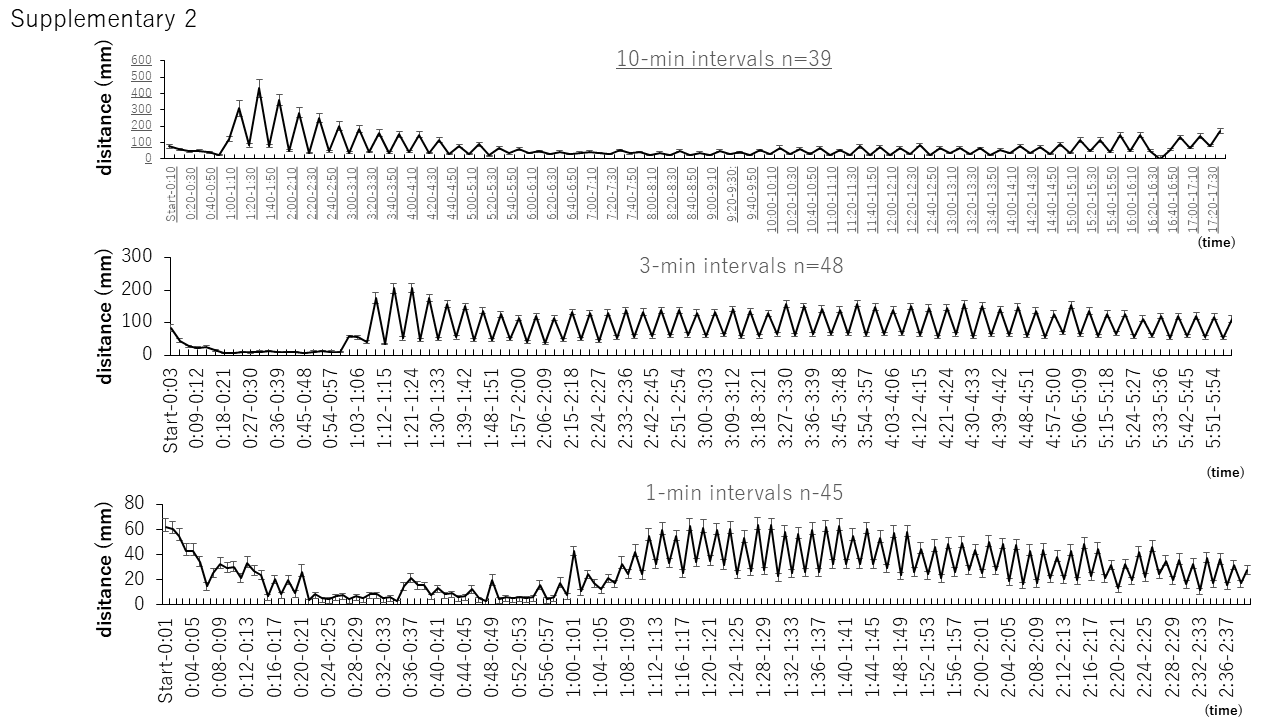

Supplement: Supplementary file 1 [file biomedicines-09-00884-s001.zip › Figure S2.TIF]
